# Supplementary material for: Genome-wide mapping of cancer dependency genes and genetic modifiers of chemotherapy in high-risk hepatoblastoma
Source: Nat Commun. 2023 Jul 6;14:4003. doi: 10.1038/s41467-023-39717-6 (PMC10326052; doi:10.1038/s41467-023-39717-6)
Supplement: Supplementary file 1 — Supplementary Information [file 41467_2023_39717_MOESM1_ESM.pdf]

Supplemental Information

Genome-wide mapping of oncogenic pathways and genetic modifiers of chemotherapy in high-risk hepatoblastoma

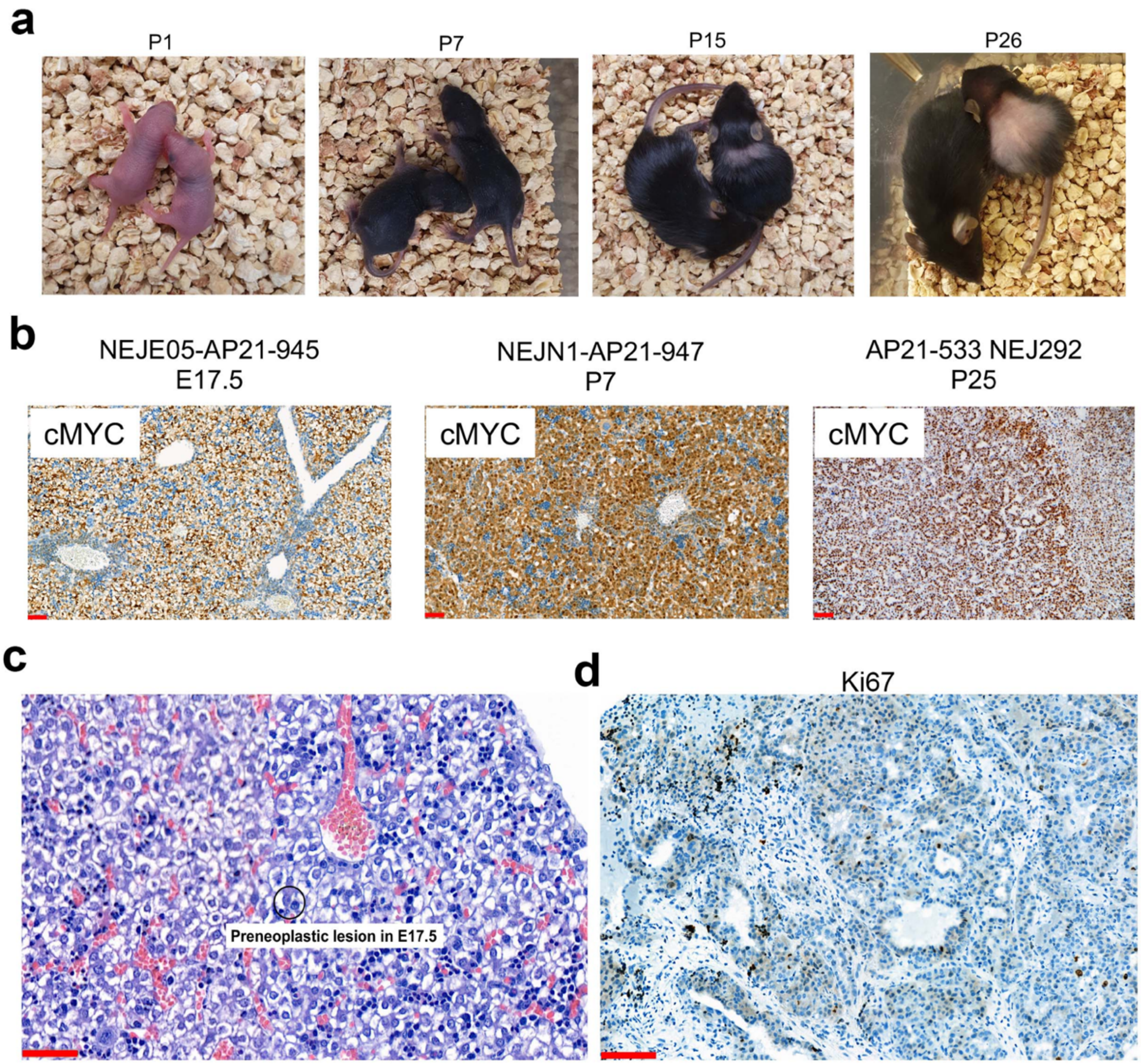

e

Pathology #ID	RS19-2057	RS22-0575	RS22-0578	RS22-0576	RS22-0577
mouse #ID	ID NEJ02	ID NEJF1	ID NEJF5	ID NEJF2	ID NEJF4
**% KI67 DAB Positive Cells**	**6.045251**	**2.948409**	**4.572658**	**5.48728**	**4.669325**
% KI67 DAB Negative Cells	93.95475	97.05159	95.427345	94.512718	95.330673
% KI67 DAB Weak Positive Cells	5.06121	1.932149	3.099779	4.03653	3.364557
% KI67 DAB Moderate Positive Cells	0.57195	0.373883	0.66581	0.72668	0.620532
% KI67 DAB Strong Positive Cells	0.412091	0.642377	0.807069	0.72407	0.684237

Supplementary Figure 1. ABC-Myc mice develop hepatoblastoma. a Paraneoplastic alopecia observed in ABC-Myc mice over time at postnatal day 1 (P1), 7 (P7), 15 (P15) and 26 (P26). **b** Immunohistochemical staining of MYC at different stages (embryonic day 17.5, postnatal day 7 and 25) of livers of ABC-Myc mice. Sample number for each image n =1. Scale bar = 25μm **c** H&E staining of ABC-Myc liver from E17.5. The circles cells indicate preneoplastic lesion of the fetal liver. Sample number for each image n =1. Scale bar = 50μm. **d** Immunohistochemical staining of ABC-Myc liver tumors with Ki67marker. Scale bar = 50μm**. e** Summary of Ki67 quantification of 5 ABC-Myc livers.

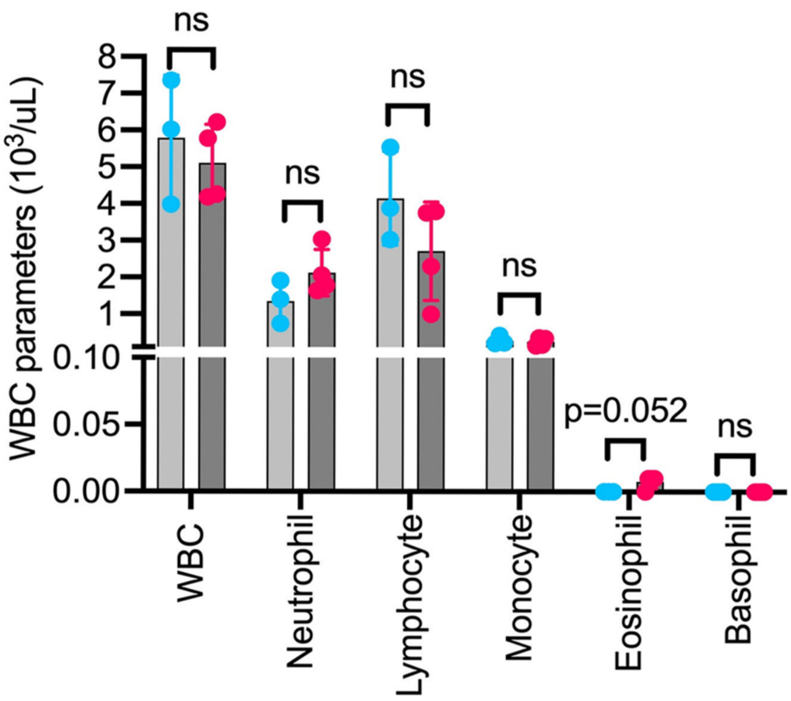

Supplementary Figure 2. Quantification of white blood cells in ABC-Myc mice. Complete blood count to determine the changes in white blood cells in plasma from normal (n= 3 biologically independent mice) and ABC-Myc (n=4 biologically independent mice) mice. Data are presented as mean ± SD. Unpaired two-sided t-test. ns = not significant.

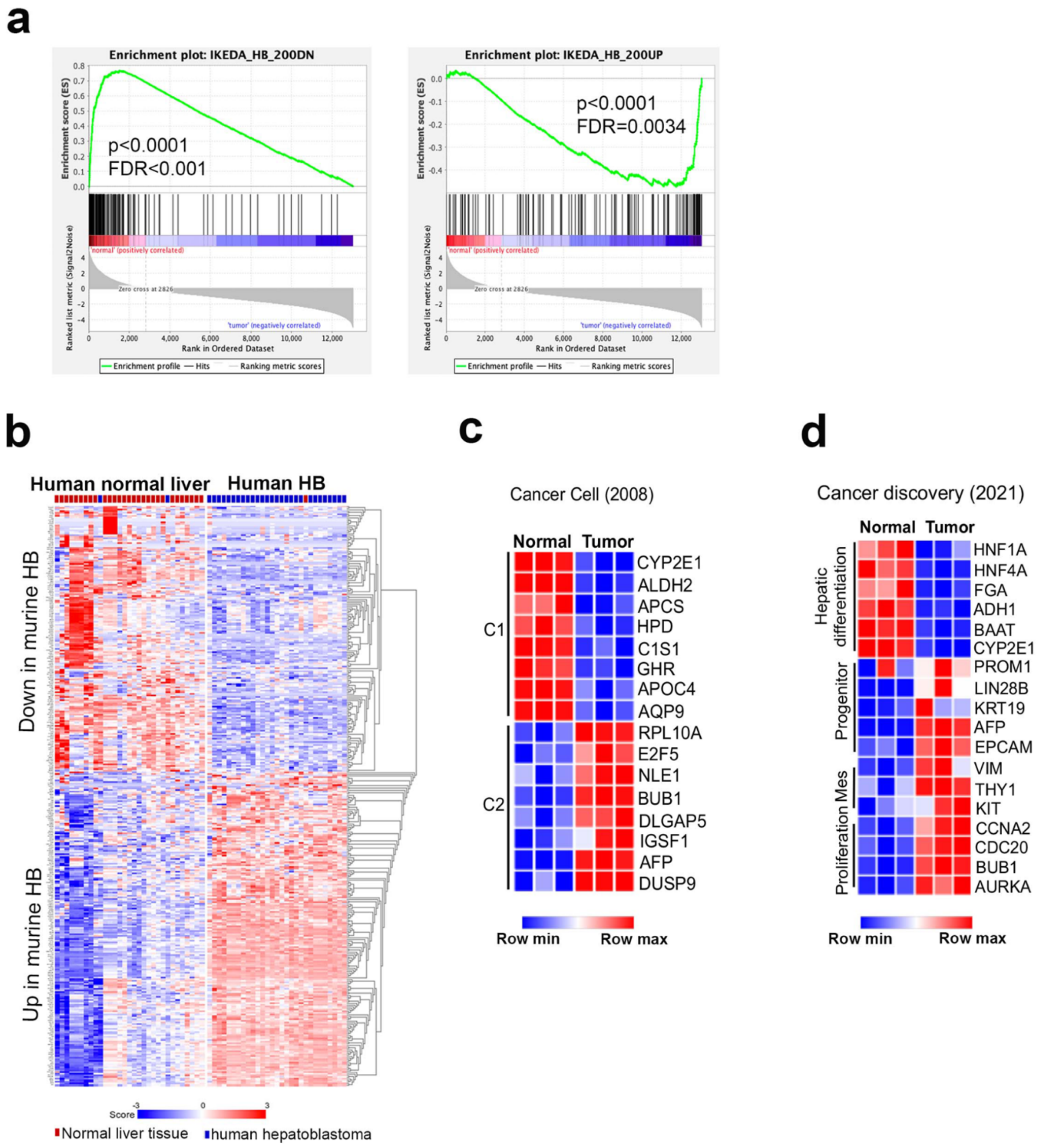

Supplementary Figure 3. The transcriptomes of ABC-Myc tumors resemble high-risk human HB.

a GSEA analysis showing that the upregulated and downregulated genes in ABC-Myc tumors are significantly associated with the top 200 genes upregulated and downregulated in human hepatoblastomas prior chemotherapy (Ikeda dataset, GSE131329)[1]. **b** Heat map showing the top differentially expressed genes in ABC-Myc tumors vs age-matched normal livers are similarly altered in human HB tumors. To determine if ABC-Myc induces transcriptomes similar to those in human hepatoblastoma, we cross-referenced our RNA-seq results for the top 500 genes upregulated and downregulated in ABC-Myc tumors with human RNA-seq dataset reported by Hooks et al (GSE104766)[2]. **c** Heatmap showing expression of C2 signature reported by Cario et al[3] in ABC-Myc tumor cells. **d** Heatmap showing high expression of 'progenitor' and 'proliferation' signatures of high-risk human hepatoblastoma (reported by et al)[4] in ABC-Myc tumor cells.

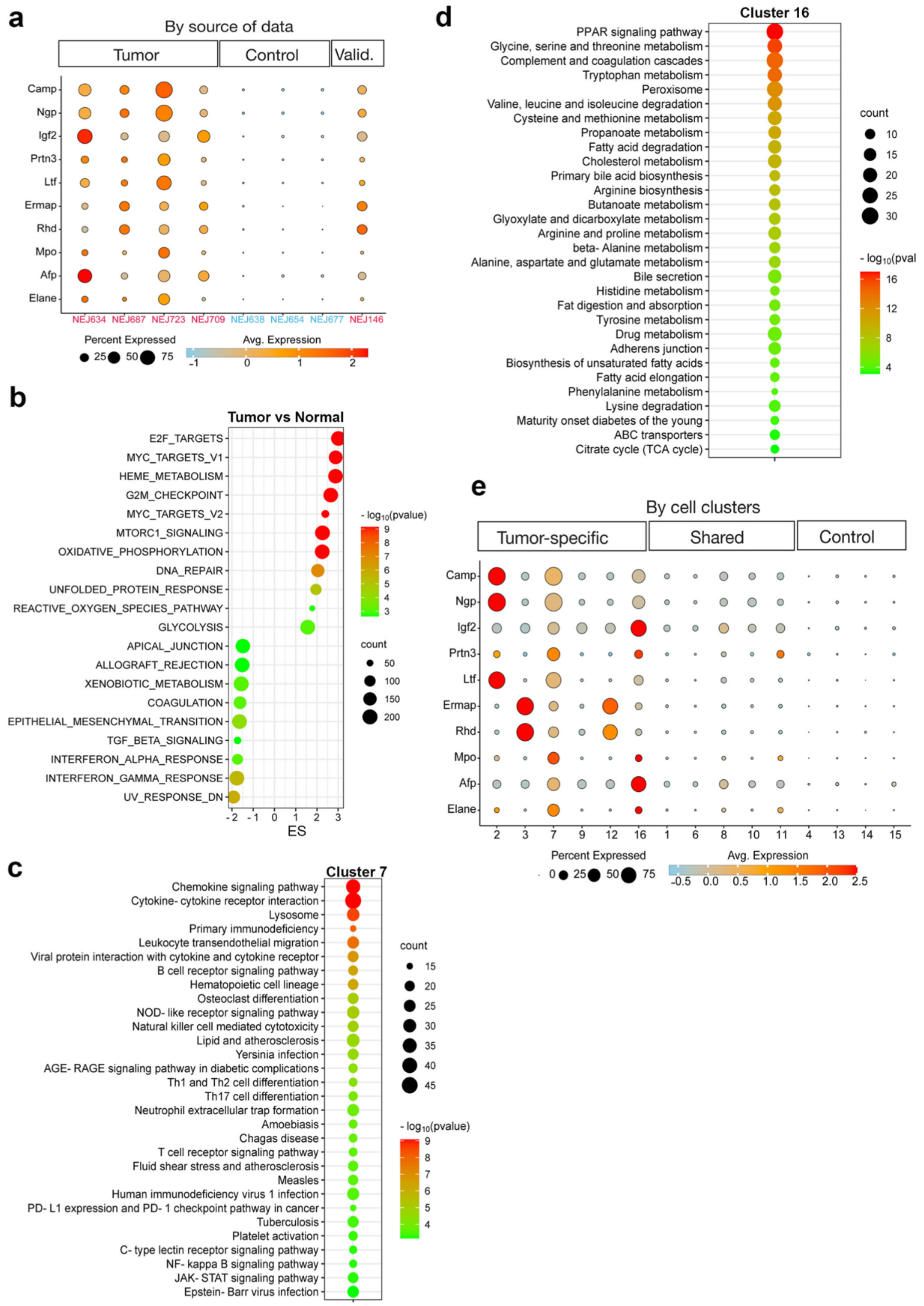

Supplementary Figure 4. scRNA-seq analysis of normal livers and ABC-Myc tumors. a Bubble plot showing the top 10 genes differentially expressed in normal livers vs ABC-Myc liver tumors. **b** Bubble plot showing the Hallmark genesets highly expressed in tumor tissues in comparison with normal livers. P value obtained from the one-sided Fisher's exact test. **c** Bubble plot showing the KEGG pathways highly expressed in cluster 7 cells. P value obtained from the one-sided Fisher's exact test. **d** Bubble plot showing the KEGG pathways highly expressed in cluster 16 cells. P value obtained from the one-sided Fisher's exact test. **e** Bubble plot showing the top 10 genes expressed in 15 clusters of cells.

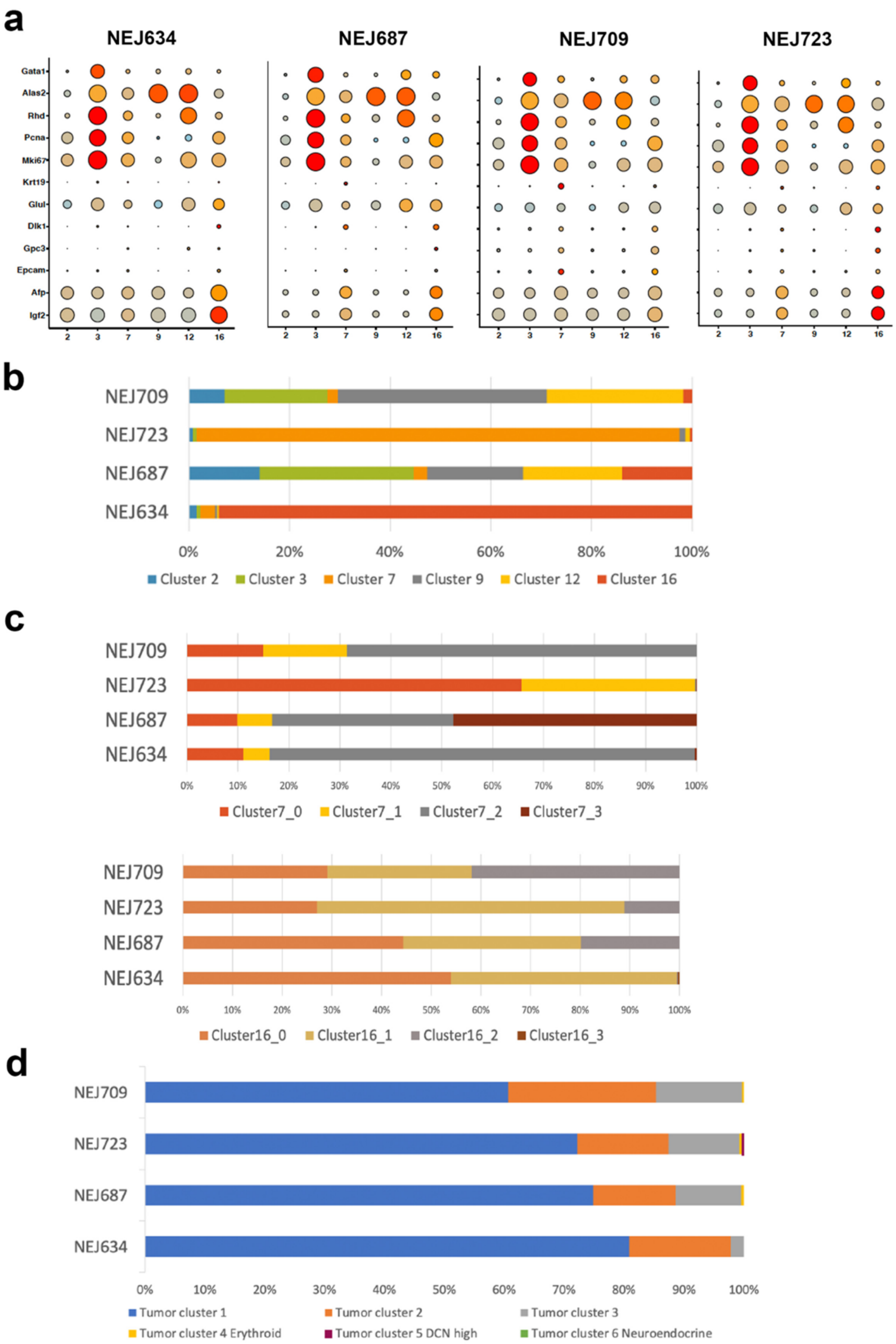

Supplementary Figure 5. scRNA-seq analysis of tumor cell heterogeneity. a Bubble plot showing the differentially expressed genes in each cluster (2, 3, 7, 9, 12, 16) of each individual ABC-Myc tumors (NEJ634, NEJ687, NEJ709, NEJ723). **b** The percentages of clusters 2, 3, 7, 9, 12 and 16 in each tumor sample, demonstrating both intra-tumoral and inter-tumoral heterogeneity. **c** The percentages of subclusters in clusters 7 and 16 of each tumor sample showing significant variation in sub-cluster proportion across tumor cells ($P < 0.0005$). **d** The percentages of human hepatoblastoma clusters (Song, et al)[5] in each tumor sample showing significant intra-tumoral heterogeneity (different tumor classes in individual tumors) and inter-tumoral heterogeneity (different composition of various tumor cell types). There is a significant variation of proportion of tumor cell types among the four samples (Chi square test: $P = 0.0005$).

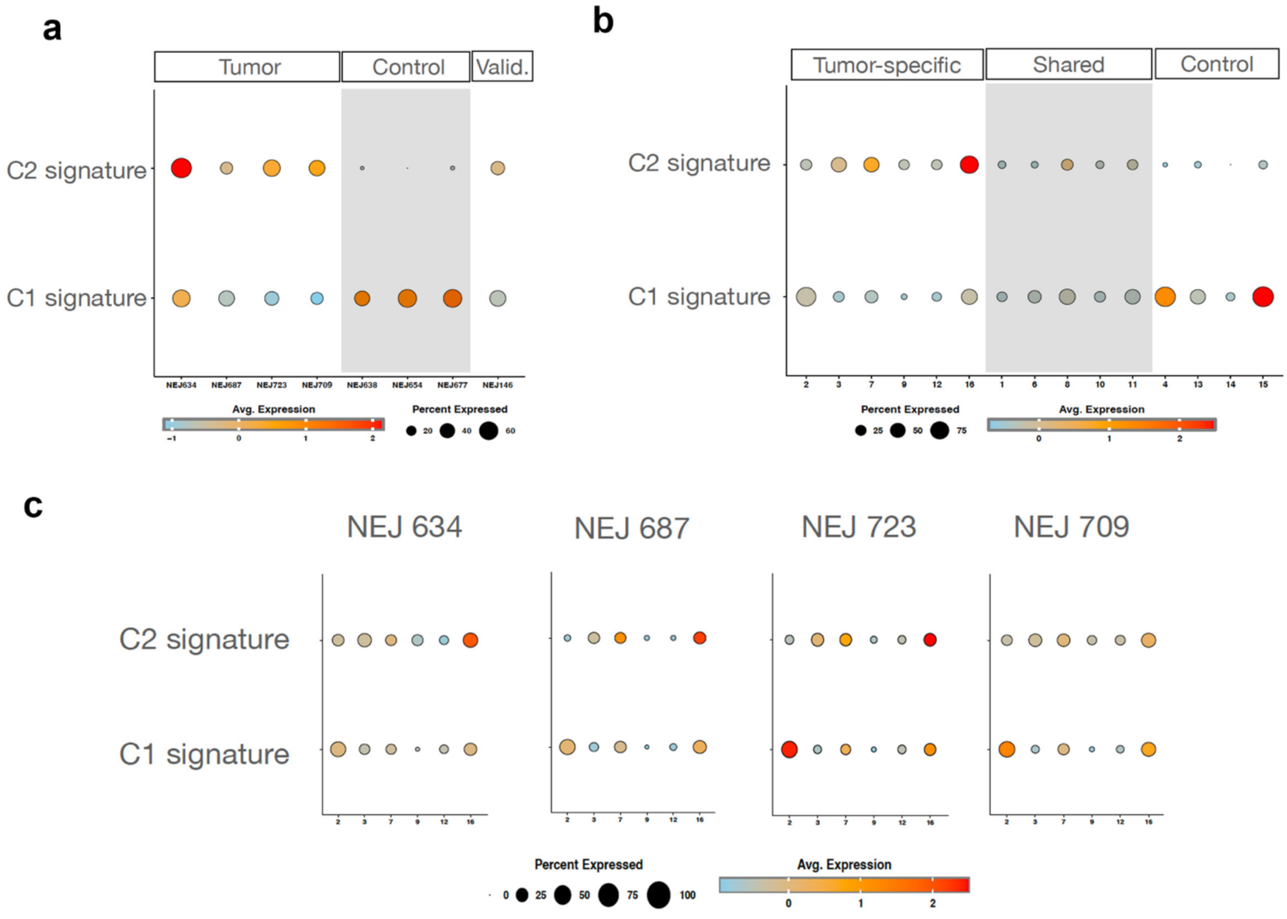

Supplementary Figure 6. Co-existence of C1 and C2 signatures in ABC-Myc tumors.
a Bubble plot of expression of C1 and C2 gene signatures in tumor and normal liver samples. **b** Bubble plot of expression of C1 and C2 gene signatures in each cluster that is tumor-specific, normal liver-specific and shared in both. **c** Bubble plot of expression of C1 and C2 gene signatures in tumor-specific clusters.

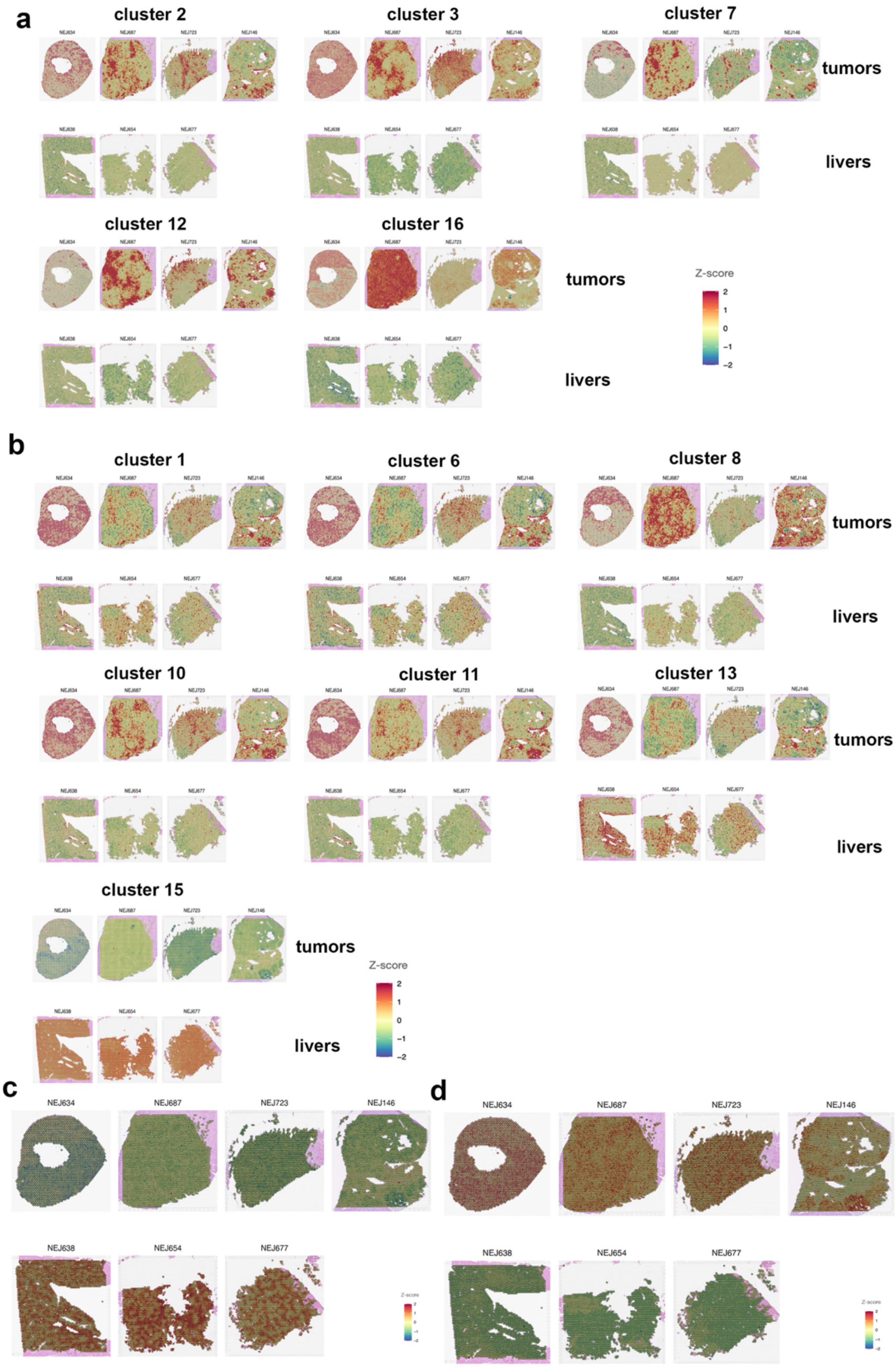

Supplementary Figure 7. Spatial transcriptomics analysis of 16 clusters of genes. a Spatial expression of tumor related clusters 2, 3, 7, 12, and 16 in in tumor tissues (NEJ634, NEJ687, NEJ723, NEJ146) and normal livers (NEJ638, NEJ654, NEJ677). **b** Spatial expression of shared and normal liver related clusters 1, 6, 8, 10, 11, 13 and 15 in in tumor tissues (NEJ634, NEJ687, NEJ723, NEJ146) and normal livers (NEJ638, NEJ654, NEJ677). **c** The spatial expression of C1 gene signature in tumor (top) and normal liver (bottom) samples. d. The spatial expression of C2 gene signature in tumor (top) and normal liver (bottom) samples.

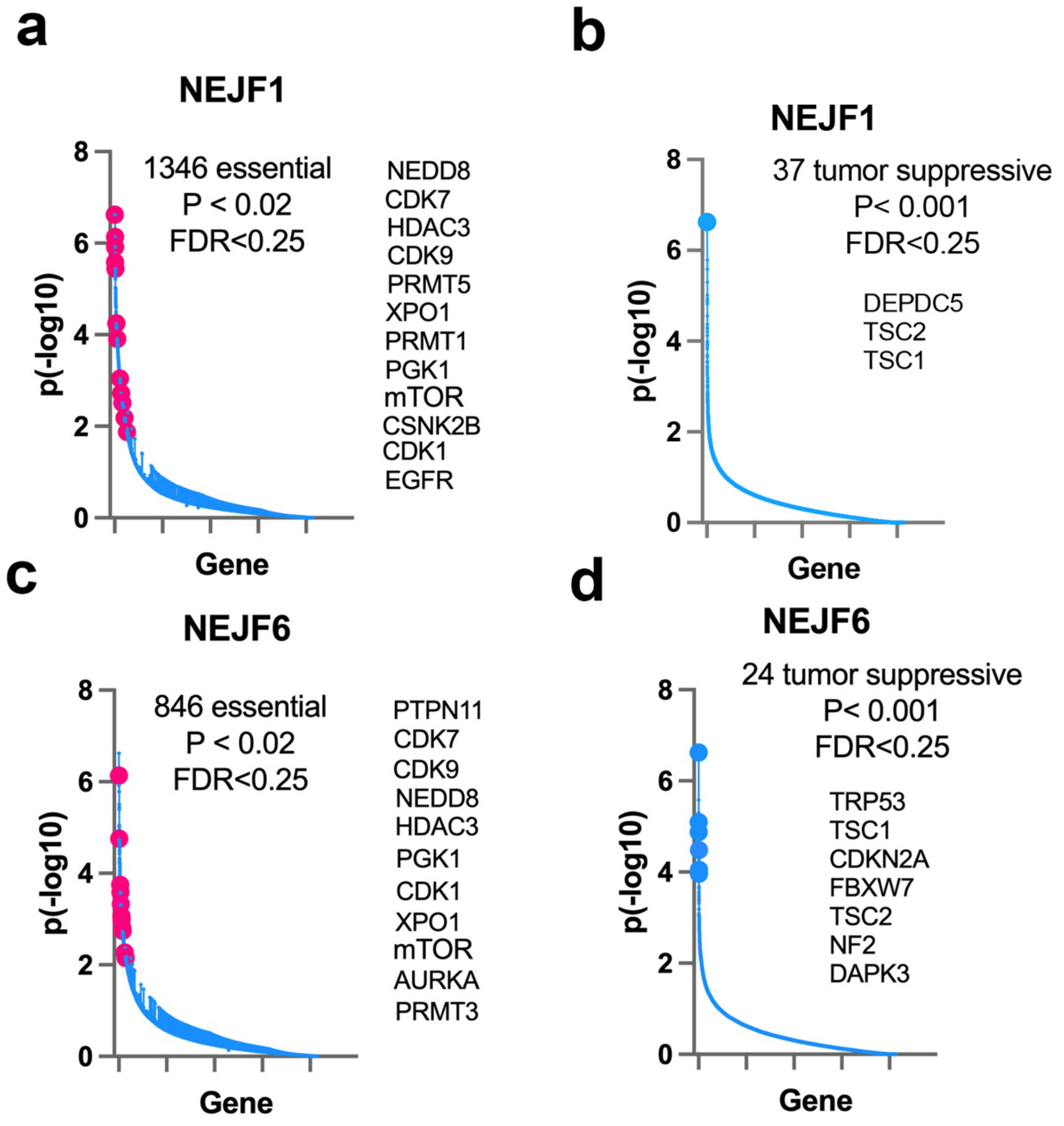

Supplementary Figure 8. CRISPR screening of cancer dependency genes in ABC-Myc cell lines NEJF1 and NEJF6.

a, b Cancer essential genes and tumor suppressors identified in NEJF1 cell line with FDR cutoff <0.25. X-axis represents the total gene number. Y axis represents the p value in -log10. The listed genes are highlighted in the graph. P value obtained by permutation test and FDR calculated from the empirical permutation p-values using the Benjamini-Hochberg procedure by MAGeCK. **c, d** Cancer essential genes and tumor suppressors identified in NEJF6 cell line with FDR cutoff <0.25. X-axis represents the total gene number. Y axis represents the p value in -log10. The listed genes are highlighted in the graph. P value obtained by permutation test and FDR calculated from the empirical permutation p-values using the Benjamini-Hochberg procedure by MAGeCK.

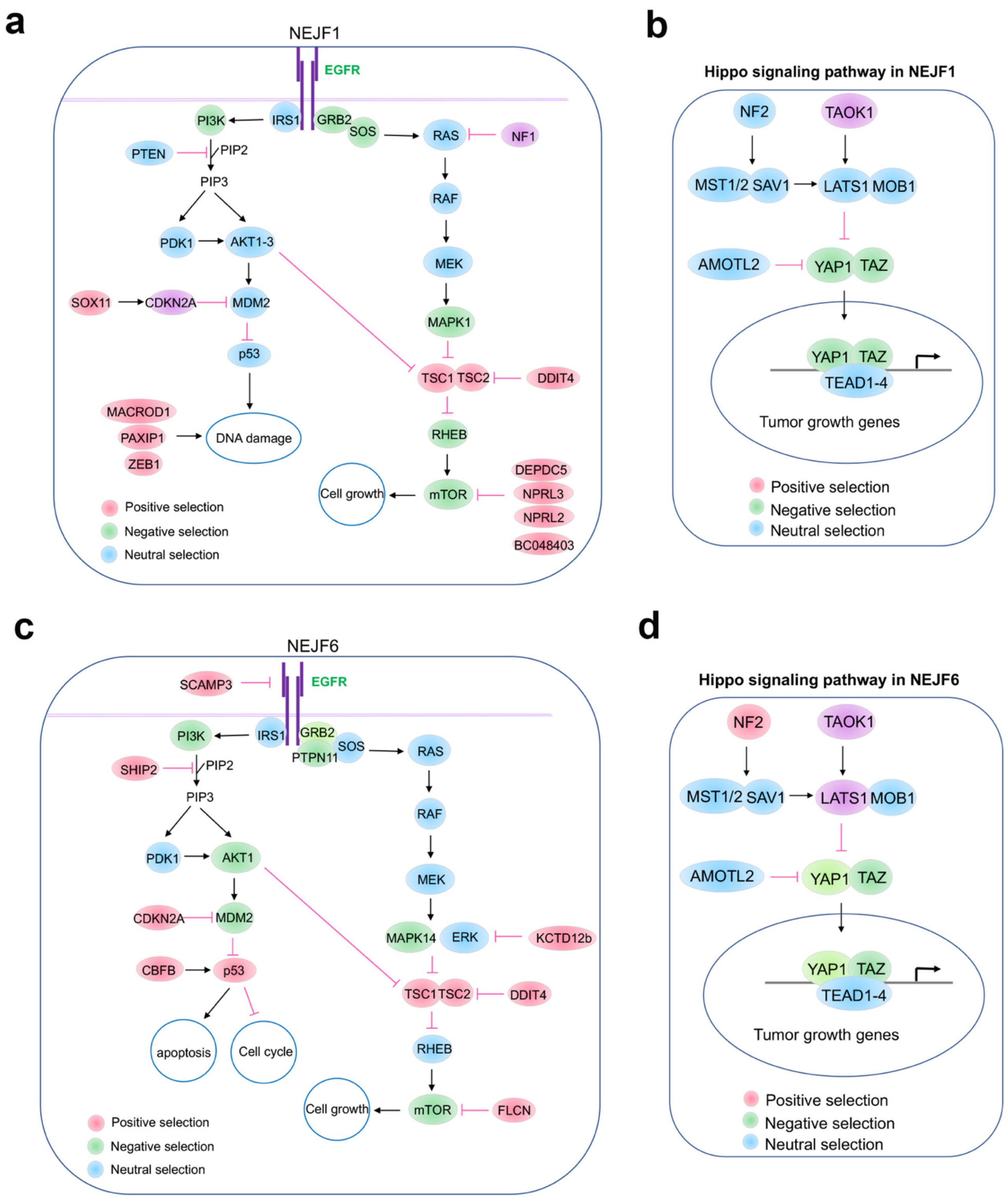

Supplementary Figure 9. Conserved oncogenic pathways in NEJF1 and NEJF6.

a Canonical cancer pathways enriched in genes identified by CRISPR screen in NEJF1 cell line. **b** Hippo signaling pathway enriched in genes identified by CRISPR screen in NEJF1 cell line. **c** Canonical cancer pathways enriched in genes identified by CRISPR screen in NEJF6 cell line. **d** Hippo signaling pathway enriched in genes identified by CRISPR screen in NEJF6 cell line.

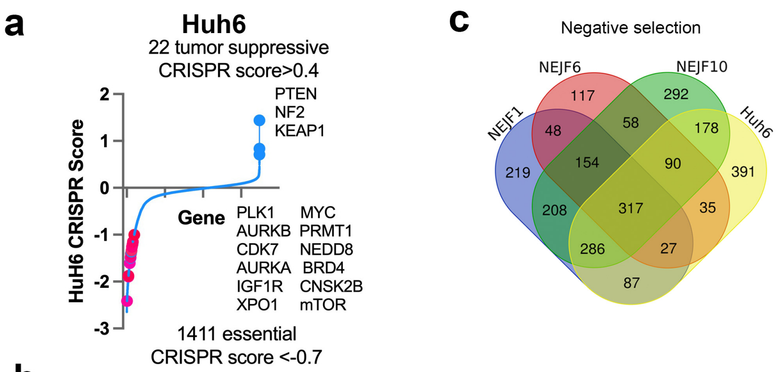

b

Gene Set Name	Description	p-value	FDR q-value
CAIRO_HEPATOBLASTOMA_CLASSES_UP	Genes up-regulated in robust Cluster 2 of hepatoblastoma samples compared to those in the robust Cluster 1	1.33E-105	2.23E-102
FISCHER_DREAM_TARGETS	Target genes of the DREAM complex.	9.93E-75	6.69E-72
WONG_EMBRYONIC_STEM_CELL_CORE	The 'core ESC-like gene module': genes coordinately up-regulated in a compendium of mouse embryonic stem cells (ESC)	2.07E-74	1.16E-71
BERENJENO_TRANSFORMED_BY_RHOA_UP	Genes up-regulated in NIH3T3 cells (fibroblasts) transformed by expression of contitutively active (Q63L) form of RHOA	1.59E-65	6.69E-63
MARTENS_TRETINOIN_RESPONSE_DN	Genes down-regulated in NB4 cells (acute promyelocytic leukemia, APL) in response to tretinoin [PubChem=444795]	6.15E-60	1.88E-57
DANG_BOUND_BY_MYC	Genes whose promoters are bound by MYC [GeneID=4609], according to MYC Target Gene Database.	7.39E-56	2.07E-53
FEVR_CTNNB1_TARGETS_DN	Genes down-regulated in intestinal crypt cells upon deletion of CTNNB1 [GeneID=1499].	1.24E-55	3.22E-53
LEE_BMP2_TARGETS_DN	Genes down-regulated in uterus upon knockout of BMP2 [GeneID=650].	3.28E-55	7.37E-53
BENPORATH_MYC_MAX_TARGETS	Set 'Myc targets2': targets of c-Myc [GeneID=4609] and Max [GeneID=4149] identified by ChIP on chip in a Burkitt's lymphoma	3.24E-53	6.42E-51
LOPEZ_MBD_TARGETS	Genes up-regulated in HeLa cells after simultaneus knockdown of all three MBD (methyl-CpG binding domain) proteins	5.77E-49	9.71E-47

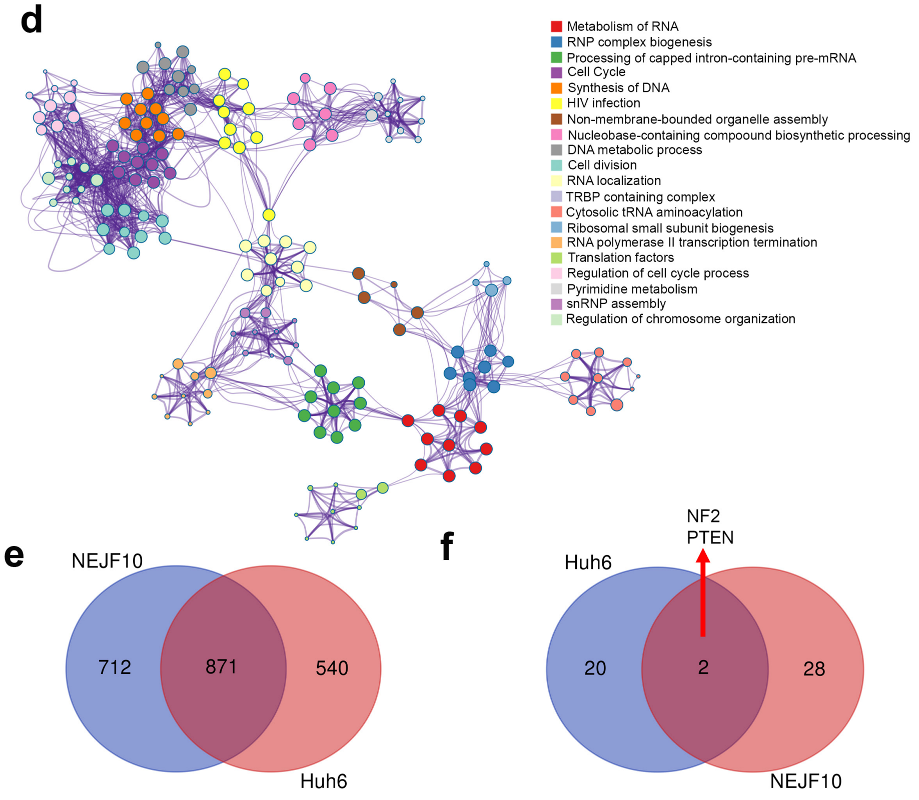

Supplementary Figure 10. CRISPR screening of cancer dependency genes in Huh6.
a Plot showing cancer essential genes (CRISPR score cutoff <-0.7) and tumor suppressors (CRISPR score cutoff > 0.4) identified in Huh6 cell line. X-axis represents the total gene number. Y axis represents the CRISPR score. The listed genes are highlighted in the graph. The CRISPR data was downloaded from DepMap program (depmap.org). **b** Pathway enrichment analysis of cancer dependency genes identified in Huh6 cell line by using GSEA and CGP (chemical and genetic pertubations) dataset. **c** Venn analysis of essential genes (negative selection) identified from NEJF1, NEJF6, NEJF10 and Huh6. **d** Network analysis of pathways using Metascape for the essential genes identified in Huh6 cells. **e** Venn analysis of essential genes (negative selection) identified from NEJF10 and Huh6. **f** Venn analysis of essential genes (positive selection) identified from NEJF10 and Huh6.

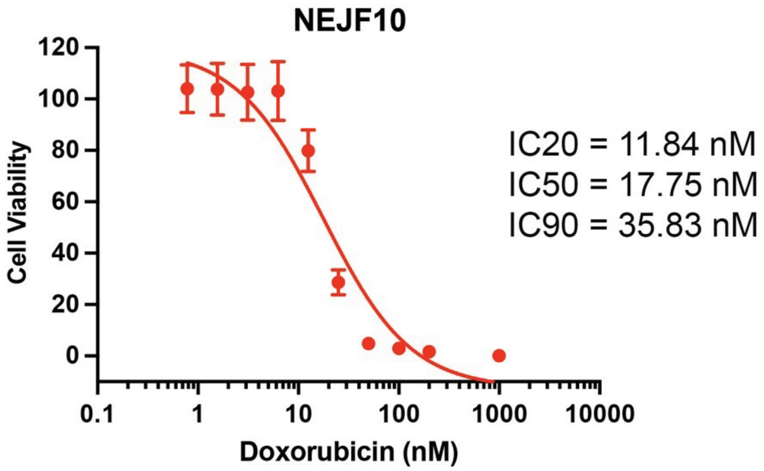

Supplementary Figure 11. Doxorubicin dose test in NEJF10 cells.
Plot showing the cell viability curve assessed by Prestoblue assay. NEJF10 cells were treated with different concentrations (n=8 for each concentration) of doxorubicin in 96-well plate for 4 days. Since doxorubicin has a very narrow therapeutic window at the nM level, we decided to reduce the doses for IC_{20} and IC_{90} by 5 nM to become ~ 5 nM for IC_{20} and ~ 30 nM for IC_{90} for a 3-week treatment in CRISPR screening. Data are presented as mean ± SD (n=8).

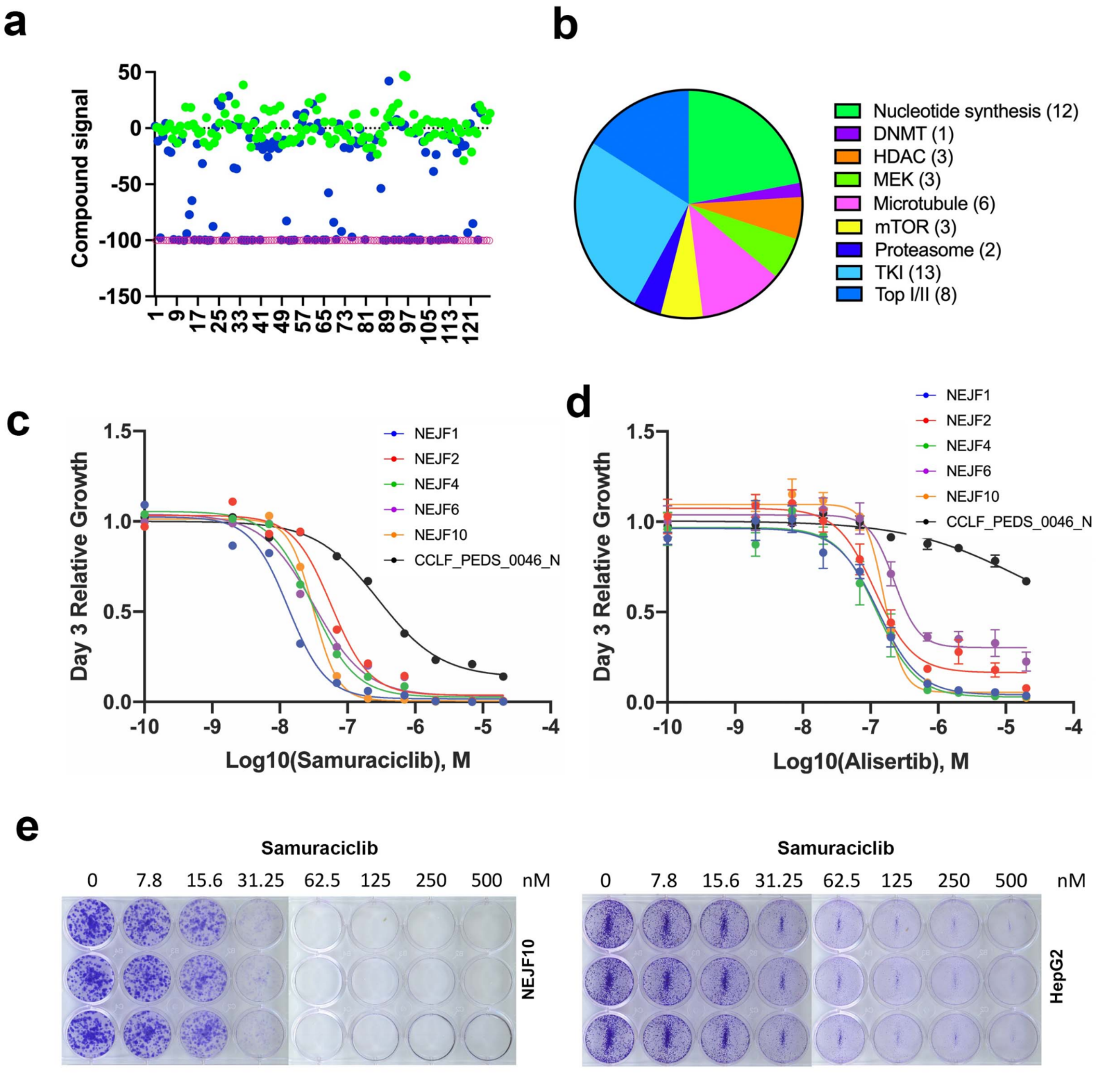

Supplementary Figure 12. Drug screening of ABC-Myc cell lines.

a Relative cell viability signal of NEJF10 cells treated with 0.7uM-2uM compounds and controls. Green color indicates DMSO as negative control. Purple color indicates 17-DMAG as positive control. Blue color indicates the 125 compounds. **b** Classes of tested compounds that exhibit anticancer activity >50% of positive control. **c, d** Cell viability assay of CDK7 inhibitor, Samuraciclib (c), and AURKA inhibitor, Alisertib (d), for ABC- Myc cell lines NEJF1, NEJF2, NEJF4, NEJF6, NEJF10 and noncancerous fibroblast cell line CCLF_PEDS_0046_N. **e** Colony formation assay to determine the effect of Samuraciclib on NEJF10 and HepG2 cells, which were treated with indicated concentrations of Samuraciclib for 7 days (n =3 biologically independent samples). This was repeated only once.

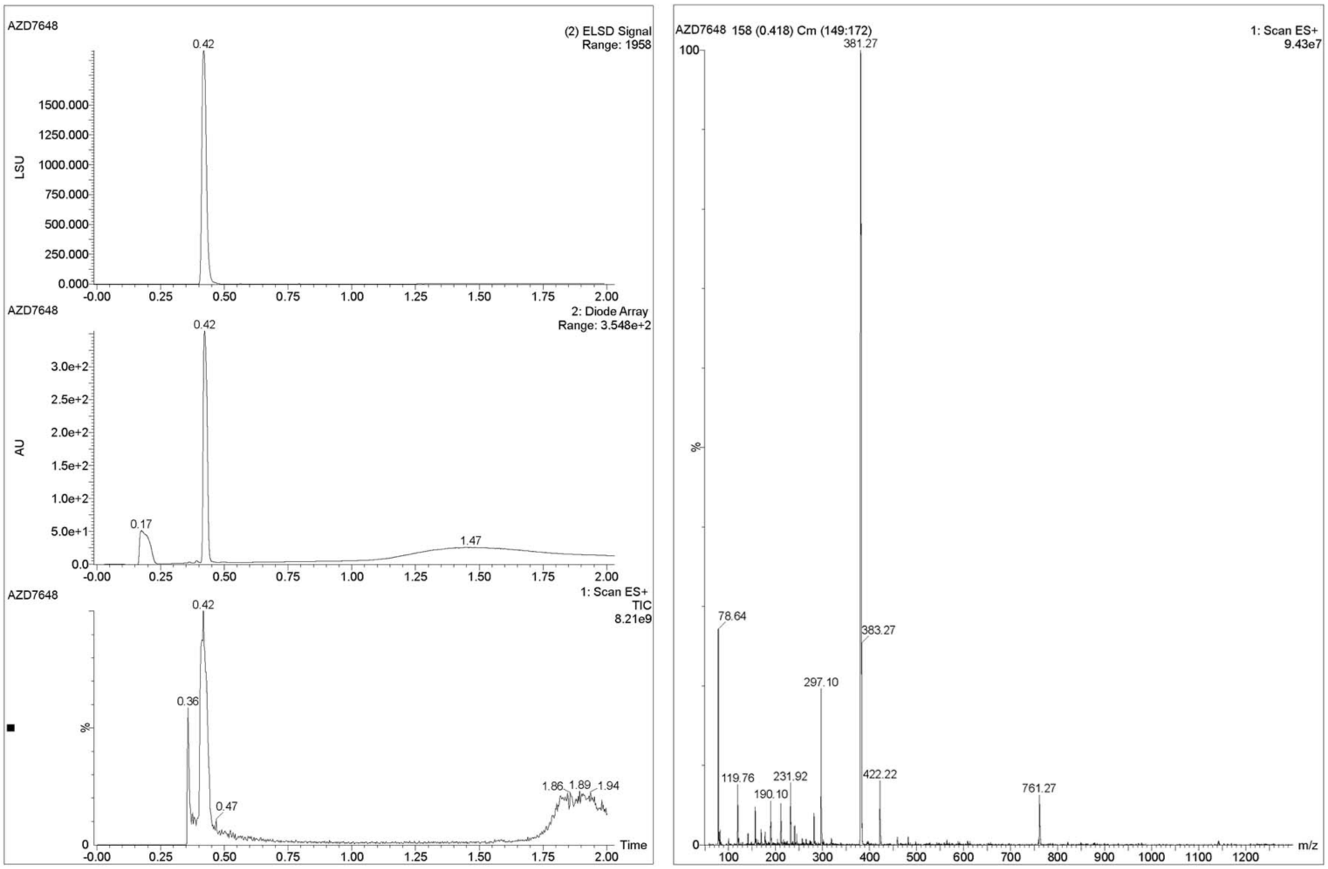

Supplementary Figure 13. Validation of AZD7648.

The purity of AZD7648 is verified by Waters UPLC-MS system (Acquity PDA detector, SQ detector and UPLC BEH-C18 column). The mass spectrometer was acquired using MassLynx v. 4.1. The chromatographic conditions are as follows: flow rate: 1.0 mL/min, sample injection volume: 2 μL, column temperature: 55 °C, mobile phase: 0.1% formic acid in CH_3CN and H_2O.

a

		Doxorubicin (nM)				
		0	7.5	15	30	60
AZD7648 (µM)	0.0	100.00	73.45	64.04	23.21	7.75
	0.1	95.46	63.31	43.29	10.28	6.29
	0.3	96.01	65.77	35.89	7.32	6.81
	1.0	97.68	25.97	11.17	7.07	7.17
	3.0	93.36	17.23	8.49	7.01	6.71

Percentage over bliss score						
		Doxorubicin (nM)				
		0	7.5	15	30	60
AZD7648 (µM)	0					
	0.1		6.81	17.84	11.88	1.11
	0.3		4.74	25.59	14.96	0.63
	1		45.77	51.38	15.60	0.40
	3		51.34	51.29	14.65	0.53

b

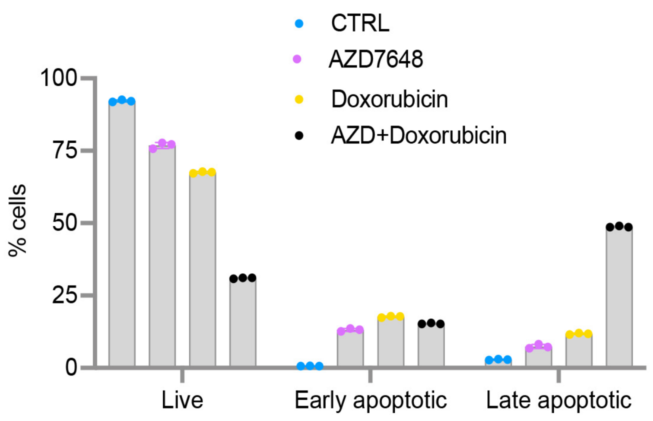

c

		Doxorubicin (nM)				
		0	5	25	125	625
AZD7648 (µM)	0	100.00	78.92	62.68	50.58	38.74
	0.1	97.61	77.38	59.41	49.95	22.90
	0.3	97.08	73.58	58.13	51.16	23.11
	1	98.67	74.04	54.18	49.91	23.78
	3	99.20	75.19	57.99	49.04	25.85

Percentage over bliss score						
		Doxorubicin (nM)				
		0	5	25	125	625
AZD7648 (µM)	0					
	0.1		-0.34	1.77	-0.58	14.91
	0.3		4.19	2.72	-2.06	14.49
	1		4.35	7.66	0.00	14.44
	3		3.42	4.19	1.14	12.58

d

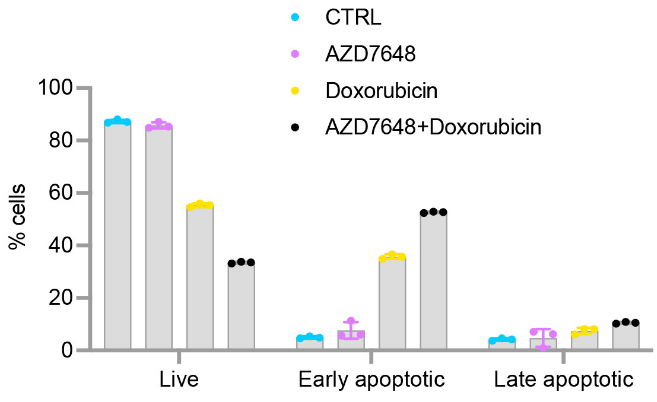

Supplementary Figure 14. Enhanced anticancer activity by combination of Doxorubicin with AZD7648. **a** Bliss index for AZD7648 (μM) and doxorubicin (nM) treatment of NEJF10 (top panel). The percentage over bliss score (bottom panel) indicates the degree of synergy. Positive scores indicate synergy, negative scores indicate antagonism. **b** Quantification of live, early apoptotic and late apoptotic cells after FACS analysis of NEJF10 treated with doxorubicin and AZD7648 for 48 hours. Data presented as scatter dot plot with mean ± SD (n =3 biologically independent samples). **c, d** Similar experiments performed using HepG2 cells. Data in d presented as scatter dot plot with mean ± SD (n =3 biologically independent samples).

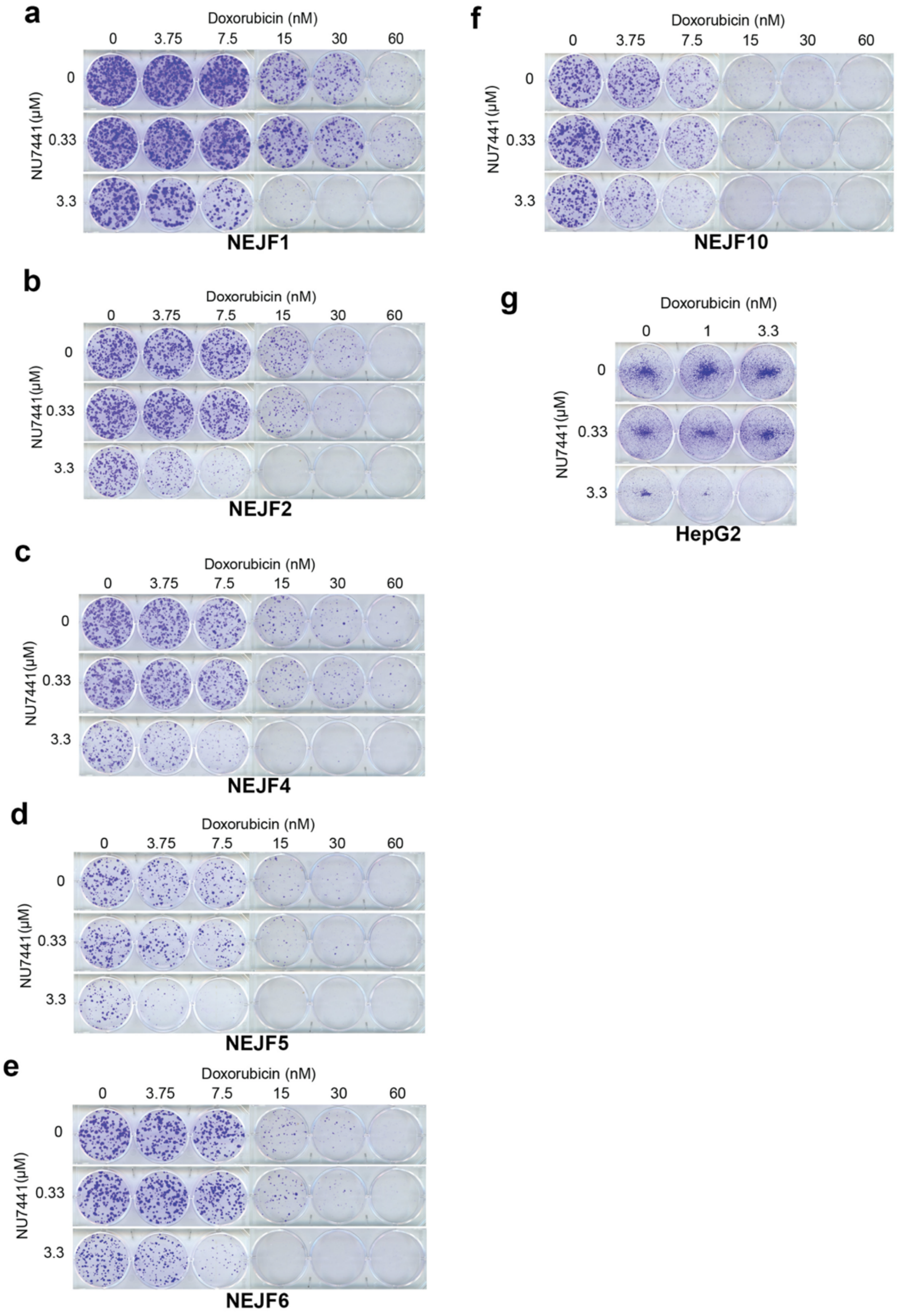

Supplementary Figure 15. Synergistic effect of Doxorubicin and NU7441. a NEJF1(500 cells per well) cells were treated with Nu7441 (0, 0.33, and 3.3 μM). Doxorubicin (0, 3.75, 7.5,15,30 and 60 nM) was added 1 hour post Nu7441 treatment. The culture medium, Nu7441 and doxorubicin were changed every 2-3 days. 7 days later, cells were washed with Dulbecco's phosphate buffered saline without calcium or magnesium and fixed with 4% formaldehyde in PBS for 20 minutes. Once formaldehyde was removed, cells were stained with 0.1% crystal violet for 1 hour. Plates were rinsed with water and imaged. **b** Same procedure as done in **a** for NEJF2. **c** Same procedure as done in **a** for NEJF4. **d** Same procedure as done in **a** for NEJF5. **e** Same procedure as done in **a** for NEJF6. **f** Same procedure as done in **a** for NEJF10 although it was stained after a 4-day treatment. **g** Same procedure as done in **a** for HepG2 (10000 cells per well) for a 12-day treatment. **a-g** are repeated by two independent experiments.

Supplementary Table 1. All possible genotypes are recovered at the expected Mendelian ratio.

Breeding strategy	Alb-Cre$^{cre/wt}$::CAG-MYC$^{wt/wt}$		Alb-Cre$^{cre/wt}$::CAG-MYC$^{myc/wt}$		Alb-Cre$^{wt/wt}$::CAG-MYC$^{myc/wt}$		Alb-Cre$^{wt/wt}$::CAG-MYC$^{wt/wt}$	
	Male	Female	Male	Female	Male	Female	Male	Female
Alb-Cre$^{cre/cre}$ x CAG-MYC $^{myc/myc}$			9	12				
Alb-Cre$^{cre/cre}$ x CAG-MYC $^{myc/wt}$	7	6	4	4				
Alb-Cre$^{cre/wt}$ x CAG-MYC $^{myc/myc}$			10	8	9	8		
Alb-Cre$^{cre/wt}$ x CAG-MYC $^{myc/wt}$	5	4	3	3	5	6	4	6

Supplementary Table 2. ABC-Myc hepatoblastomas recapitulate the metabolic pathways in human hepatoblastomas.

KEGG pathway (up in mouse liver tumor)	GSEA score	GSEA p_value	up in human HB
Cell_cycle	-2.24	0	2.5E-19
DNA_replication	-2.3	0	9.8E-09
Nucleotide_excision_repair	-1.84	0	0.000085
Pyrimidine_metabolism	-1.83	0	0.00034
Mismatch_repair	-2.26	0	0.00049
Spliceosome	-2.43	0	0.0012
Oocyte_meiosis	-1.77	0	0.0016
Ribosome_biogenesis_in_eukaryotes	-2.89	0	0.03
Purine_metabolism	-1.65	0.001	0.01
p53_signaling_pathway	-1.56	0.007	0.0066
Basal_transcription_factors	-1.58	0.01	0.00015
Ubiquitin_mediated_proteolysis	-1.38	0.016	0.0011
Glycosylphosphatidylinositol_GPI__anchor_biosynthesis	-1.12	0.312	0.01
**KEGG pathway (down in mouse liver tumor)**	**GSEA score**	**GSEA p_value**	**down in human HB**
Complement_and_coagulation_cascades	3.28	0	6.7E-20
Tryptophan_metabolism	2.95	0	5.1E-09
Valine__leucine_and_isoleucine_degradation	2.48	0	5.2E-09
Retinol_metabolism	3.5	0	0.000000043
Metabolism_of_xenobiotics_by_cytochrome_P450	3.08	0	0.000000091
Propanoate_metabolism	1.79	0	0.00000021
Glycine__serine_and_threonine_metabolism	1.65	0	0.000006
Peroxisome	3.46	0	0.0000061
Butanoate_metabolism	2.2	0	0.0000086
PPAR_signaling_pathway	3.06	0	0.000023
Pyruvate_metabolism	1.69	0	0.000029
Steroid_hormone_biosynthesis	2.96	0	0.000065
Fatty_acid_degradation	2.46	0	0.000073
Bile_secretion	3.07	0	0.00017
Drug_metabolism___other_enzymes	2	0.008	6.5E-10

Supplementary Table 3. ABC-Myc tumors recapitulate C2 class of human hepatoblastoma.

Array id	Compound Covariate Predictor	Linear Discriminant Analysis	1-Nearest Neighbor	3-Nearest Neighbors	Nearest Centroid	Support Vector Machines	Bayesian Compound Covariate Predictor
E3-Normal	1	1	1	1	1	1	1
A3-Normal	1	1	1	1	1	1	1
B3-Normal	1	1	1	1	1	1	1
C3-HB	2	2	2	2	2	2	2
D3-HB	2	2	2	2	2	2	2
F3-HB	2	2	2	2	2	2	2

The sixteen-gene RNA-seq data were used to assign tumors to HB molecular subclasses by using the method described by Cairo et al[3].

Supplementary Table 4. Average percentage of UMI in each cluster of cells.

Source	Cluster	Average percent of HB UMI
Shared	cluster1 (NK/T cells)	2.86
	cluster6 (B cells)	1.86
	cluster8 (Macrophage)	3.26
	cluster10 (Neutrophils)	3.04
	cluster11 (Monocytes)	2.26
Normal	cluster4 (Hepatocytes I)	1.30
	cluster13 (Endothelial)	1.15
	cluster14 (Erythroid)	87.58
	cluster15 (Hepatocytes II)	0.68
Tumor	**cluster2 (HB associated Pro-myelocyte)**	2.23
	**cluster3 (HB associated proliferating Erythroid)**	33.65
	**cluster7 (Afp^{weak} Tumor Cluster)**	30.73
	**cluster9 (HB associated Erythroid II)**	83.57
	**cluster12 (HB associated Erythroid III)**	62.50
	**cluster16 (Afp^{strong} Tumor Cluster)**	6.12

Supplementary References

1 Kanawa M., H. E., Kawashima K., Hiyama K., Ikeda K., Morihara N., Kurihara S., Fukazawa T., Ueda Y. Gene expression profiling in hepatoblastoma cases of the Japanese study group for pediatric liver tumors-2 (JPLT-2) trial. *Eur. J. Mol. Cancer* **1**, 1-8 (2019). https://doi.org:DOI:10.31487/J.EJMC.2018.01.003

2 Hooks, K. B. *et al.* New insights into diagnosis and therapeutic options for proliferative hepatoblastoma. *Hepatology* **68**, 89-102 (2018). https://doi.org:10.1002/hep.29672

3 Cairo, S. *et al.* Hepatic stem-like phenotype and interplay of Wnt/beta-catenin and Myc signaling in aggressive childhood liver cancer. *Cancer Cell* **14**, 471-484 (2008). https://doi.org:10.1016/j.ccr.2008.11.002

4 Hirsch, T. Z. *et al.* Integrated Genomic Analysis Identifies Driver Genes and Cisplatin-Resistant Progenitor Phenotype in Pediatric Liver Cancer. *Cancer Discov* **11**, 2524-2543 (2021). https://doi.org:10.1158/2159-8290.CD-20-1809

5 Song, H. *et al.* Single-cell analysis of hepatoblastoma identifies tumor signatures that predict chemotherapy susceptibility using patient-specific tumor spheroids. *Nat Commun* **13**, 4878 (2022). https://doi.org:10.1038/s41467-022-32473-z
