# Supplementary material for: Genome-wide mapping of cancer dependency genes and genetic modifiers of chemotherapy in high-risk hepatoblastoma
Source: Nat Commun. 2023 Jul 6;14:4003. doi: 10.1038/s41467-023-39717-6 (PMC10326052; doi:10.1038/s41467-023-39717-6)
Supplement: Supplementary file 3 — Description of Additional Supplementary Files [file 41467_2023_39717_MOESM3_ESM.pdf]

Description of Additional Supplementary Files

File Name: Supplementary Data 1
Description: Bulk RNA-seq for ABC-Myc liver cancer model.

File Name: Supplementary Data 2
Description: Single cell cluster summary stats.

File Name: Supplementary Data 3
Description: Cluster 7 against other tumor clusters.

File Name: Supplementary Data 4
Description: Cluster 16 against other tumor clusters.

File Name: Supplementary Data 5
Description: Murine HB NEJF10 CRISPR screen.

File Name: Supplementary Data 6
Description: Targets with inhibitors available.

File Name: Supplementary Data 7
Description: Murine HB NEJF1 CRISPR screen.

File Name: Supplementary Data 8
Description: Murine HB NEJF6 CRISPR screen.

File Name: Supplementary Data 9
Description: Venn for NEJF1, NEJF6, NEJF10 CRISPR screen.

File Name: Supplementary Data 10
Description: DepMap Huh6.

File Name: Supplementary Data 11
Description: Venn for essential genes in Huh6 and NEJF1, NEJF6, NEJF10.

File Name: Supplementary Data 12
Description: IC20 doxorubicin CRISPR screen in NEJF10.

File Name: Supplementary Data 13
Description: IC90 doxorubicin CRISPR screen in NEJF10.
