# Supplementary material for: Genome-wide mapping of cancer dependency genes and genetic modifiers of chemotherapy in high-risk hepatoblastoma
Source: Nat Commun. 2023 Jul 6;14:4003. doi: 10.1038/s41467-023-39717-6 (PMC10326052; doi:10.1038/s41467-023-39717-6)
Supplement: Supplementary file 17 — Reporting Summary [file 41467_2023_39717_MOESM17_ESM.pdf]

nature portfolio

Corresponding author(s): Jun Yang, Xiang Chen

Last updated by author(s): 1/31/2023

Reporting Summary

Nature Portfolio wishes to improve the reproducibility of the work that we publish. This form provides structure for consistency and transparency in reporting. For further information on Nature Portfolio policies, see our Editorial Policies and the Editorial Policy Checklist.

Statistics

For all statistical analyses, confirm that the following items are present in the figure legend, table legend, main text, or Methods section.

n/a	Confirmed	
☐	☒	The exact sample size (*n*) for each experimental group/condition, given as a discrete number and unit of measurement
☐	☒	A statement on whether measurements were taken from distinct samples or whether the same sample was measured repeatedly
☐	☒	The statistical test(s) used AND whether they are one- or two-sided<br>*Only common tests should be described solely by name; describe more complex techniques in the Methods section.*
☒	☐	A description of all covariates tested
☒	☐	A description of any assumptions or corrections, such as tests of normality and adjustment for multiple comparisons
☐	☒	A full description of the statistical parameters including central tendency (e.g. means) or other basic estimates (e.g. regression coefficient) AND variation (e.g. standard deviation) or associated estimates of uncertainty (e.g. confidence intervals)
☐	☒	For null hypothesis testing, the test statistic (e.g. *F*, *t*, *r*) with confidence intervals, effect sizes, degrees of freedom and *P* value noted<br>*Give P values as exact values whenever suitable.*
☒	☐	For Bayesian analysis, information on the choice of priors and Markov chain Monte Carlo settings
☒	☐	For hierarchical and complex designs, identification of the appropriate level for tests and full reporting of outcomes
☒	☐	Estimates of effect sizes (e.g. Cohen's *d*, Pearson's *r*), indicating how they were calculated

Our web collection on statistics for biologists contains articles on many of the points above.

Software and code

Policy information about availability of computer code

Data collection	Bulk RNA-seq data were collected by Paired-end sequencing using the Illumina NovSeq with 150bp read length.<br>For scRNA-seq and spatial transcriptomics, data were collected by Cell Ranger V6 and Space ranger Software 1.2 (10X Genomics)
Data analysis	For bulk RNA-seq data analysis, Trim-Galore version v0.60, STAR v2.7, RSEM v1.31, gsea2.2.3 and MSigDB 6.2 were used. For scRNA-seq and spatial transcriptomics, data were processed by Cell Ranger Software (10X Genomics). NBID (https://github.com/chenlab-sj/nbid) was used for differential expression analysis for scRNA-seq analysis, which depends on R package SVA (3.46.0), scran (1.26.1) and edgeR (3.40.1). LCA (https://github.com/chenlab-sj/single_cell_lca) was used for scRNA-seq analysis. Seurat (4.3.0) was used for spatial transcriptomics analysis. R packages celldex (version 1.6.0), ClusterProfiler (4.4.4) and SingleR (2.0.0) were used for data annotation. Dimension reduction using tsne was performed using Rtsne (0.16). R pcakge SCpubr (1.0.4.9000), pheatmap (1.0.12), ggplot2 (3.4.0) and ggpubr (0.5.0) were used for data visualization.

For manuscripts utilizing custom algorithms or software that are central to the research but not yet described in published literature, software must be made available to editors and reviewers. We strongly encourage code deposition in a community repository (e.g. GitHub). See the Nature Portfolio guidelines for submitting code & software for further information.

Data

Policy information about availability of data

All manuscripts must include a data availability statement. This statement should provide the following information, where applicable:

- Accession codes, unique identifiers, or web links for publicly available datasets
- A description of any restrictions on data availability
- For clinical datasets or third party data, please ensure that the statement adheres to our policy

The bulk RNA-seq, scRNA-seq, and Spatial transcriptomics data have been deposited to the Gene Expression Omnibus-GEO (NCBI) as superseries. The bulk RNA-seq is under accession number SuperSeries: GSE193124. The scRNA-seq and spatiotranscriptomics GEO accession number of this SuperSeries: GSE223689,GSE194051,GSE195575. For datasets re-used in this study included GSE79084 [https://www.ncbi.nlm.nih.gov/geo/query/acc.cgi?acc=GSE79084], GSE94858 [https://www.ncbi.nlm.nih.gov/gds/?term=GSE94858], GSE133039 [https://www.ncbi.nlm.nih.gov/geo/query/acc.cgi?acc=GSE133039]. GSE131329 [https://www.ncbi.nlm.nih.gov/gds/?term=GSE131329].
Databases/datasets used in the study were the following: R2: Genomics Analysis and Visualization Platform (https://hgserver1.amc.nl/cgi-bin/r2/main.cgi), DepMap data (https://depmap.org/portal/).

Field-specific reporting

Please select the one below that is the best fit for your research. If you are not sure, read the appropriate sections before making your selection.

☒ Life sciences ☐ Behavioural & social sciences ☐ Ecological, evolutionary & environmental sciences

For a reference copy of the document with all sections, see nature.com/documents/nr-reporting-summary-flat.pdf

Life sciences study design

All studies must disclose on these points even when the disclosure is negative.

Sample size	Experiments were designed to have enough sample sizes to obtain reliable results. We estimated the sample size considering no significant variation within each group of data. The principle of using the smallest sample size possible was adopted in planning the animal experiments. We estimated the sample size in order to detect a difference in averages of 2 standard deviations at the 0.05 level of significance with an 80% power. Mouse survival curve in Figure 1c, wiltype mice n=33, ABC-MYC mice n=32, ABC-MYC:TdTomato mice n=11. Sample size in Figure 2a, normal mice n=4, ABC-myc mice n=6. Figuer 2b-c, normal liver n=3, ABC-MYC liver n=4. Sample size in in vivo efficacy in Figure 8e, vehicle mice n=6, AZD7648 mice n=7, Doxorubicin mice n=7, combination mice n=8. Sample size in in vivo efficacy in Figure 8g, vehicle mice n=5, AZD7648 mice n=5, Doxorubicin mice n=5, combination mice n=5. Sample size in in vivo efficacy in Figure 8h, vehicle mice n=4, AZD7648 mice n=4, Doxorubicin mice n=5, combination mice n=4. Carrillo-Reixach dataset sample n=66, Raymond hepatoblastoma dataset sample size n=62, López-Terrada dataset sample size n=55. For in vitro experiments, at least three independent replicates and a minimum of two biological replicates were used for each experiment to ensure the reproducibility and to perform statistical analysis.
Data exclusions	No data exclusion
Replication	Data was obtained from three technical replicates and in at least two biological replicates. Independent biological replicates are shown in all figures.
Randomization	Randomization was not relevant. All cell lines or biological samples were analyzed or treated in the same manner.
Blinding	Experiments were not blinded in order to allow the investigators to have correct identification of samples and to ensure the correct data collection. In other hand, blinding strategy was applied to computational biologists who performed bioinformatic analyses.

Reporting for specific materials, systems and methods

We require information from authors about some types of materials, experimental systems and methods used in many studies. Here, indicate whether each material, system or method listed is relevant to your study. If you are not sure if a list item applies to your research, read the appropriate section before selecting a response.

Materials & experimental systems

n/a	Involved in the study
☐	☒ Antibodies
☐	☒ Eukaryotic cell lines
☒	☐ Palaeontology and archaeology
☐	☒ Animals and other organisms
☒	☐ Human research participants
☒	☐ Clinical data
☒	☐ Dual use research of concern

Methods

n/a	Involved in the study
☒	☐ ChIP-seq
☒	☐ Flow cytometry
☒	☐ MRI-based neuroimaging

Antibodies

Antibodies used

C-MYC (Cell Signaling Technology, 5605S, RRID:AB_1903938, 1:1000), PRKDC (DNA-PK))Novus, sc57-08, RRID: AB_2809479, 1:1000), beta-actin (Sigma, A5441, RRID:AB_476744 , 1:5000) and GAPDH-HRP (Cell Signaling Technology, 3683S, RRID:AB_1642205, 1:1000) were used for western blot. Secondary horseradish peroxidase (HRP)-conjugated goat anti-mouse, Thermo Fischer Scientific Cat#31430, RRID:AB_228307,1:5000 for western blot
Secondary horseradish peroxidase (HRP)-conjugated goat anti-rabbit, Thermo Fischer Scientific Cat#31460, RRID:AB_228341, 1:5000 for western blot.

Validation

All antibodies were purchased from commercial vendors and were validated by manufactures, other studies and/or in this study. We also provided associated datasheets link as below. Please note some antibodies may have been discontinued.
C-MYC https://www.cellsignal.com/products/primary-antibodies/c-myc-d84c12-rabbit-mab/5605
PRKDC https://www.novusbio.com/products/dna-pkcs-antibody-sc57-08_nbp2-67554
beta-actin https://www.sigmaaldrich.com/US/en/product/sigma/a5316?gclid=EAIaIQobChMIzdKcp9fr9gIVXHxvBB2YFwK5EAAYASAAEgLYrfD_BwE
GAPDH https://www.cellsignal.com/products/antibody-conjugates/gapdh-14c10-rabbit-mab-hrp-conjugate/3683

Eukaryotic cell lines

Policy information about cell lines

Cell line source(s)

Human: HepG2 (ATCC, HB-8065)
Murine: NEJF1 (this manuscript)
Murine: NEJF2 (this manuscript)
Murine: NEJF4 (this manuscript)
Murine: NEJF5 (this manuscript)
Murine: NEJF10 (this manuscript)

Authentication

HepG2 was authenticated by short tandem repeat (STR) using Promega PowerPlex 16 HS System once per month.

Mycoplasma contamination

PCR-based method was used for detection of Mycoplasma with LookOut Mycoplasma PCR Detection Kit (Sigma) and JumpStart Taq DNA Polymerase (Sigma) once per month to ensure cells were mycoplasma negative.

Commonly misidentified lines
(See ICLAC register)

No commonly misidentified cell lines were used in this study.

Animals and other organisms

Policy information about studies involving animals; ARRIVE guidelines recommended for reporting animal research

Laboratory animals

Albumin-Cre (Alb-Cre) (Strain #003574), R26StopFLMYC (CAG-MYC) (Strain #020458), and CAG-tdTomato (Strain #007914) mice were obtained from the Jackson Laboratory. ABC-Myc (Alb-Cre+/wt::CAG-MYCmyc/wt) mouse model was generated by crossbreeding Alb-Cre+/+ with CAG-MYCmyc/myc mouse, or Alb-Cre+/wt with CAG-MYCmyc/myc, or Alb-Cre+/wt with CAG-MYCmyc/wt. NOD.Cg-Prkdc scid Il2rg tm1Wjl /SzJ (NSG) were provided by St Jude Animal Facility for PDX implantation. Both genders of transgenic mice were used in this study. All transgenic mice used in this study were 1 week to 1 year old depending on the experimental purpose. When used for breeding, mouse age was between 2 months to 1 year old. When used for tumorigenesis, mouse age was between 1 week and 3 months. NSG mice were aged about 5 weeks and only female NSG mice were used in PDX therapy.

Wild animals

No wild animals were used in this study

Field-collected samples

No Field-collected samples were used in this study

Ethics oversight

All experiments that involved the use of mice were performed in accordance with the guidelines outlined by the St Jude Children's Research Hospital Institutional Animal Care and Use Committee (IACUC).

Note that full information on the approval of the study protocol must also be provided in the manuscript.
